# 4-(Dimethyl­amino)pyridinium dibromido(4-bromo­phen­yl)dimethyl­stannate(IV)

**DOI:** 10.1107/S1600536808032248

**Published:** 2008-10-11

**Authors:** Chin Koon Yau, Kong Mun Lo, Seik Weng Ng

**Affiliations:** a Department of Chemistry, University of Malaya, 50603 Kuala Lumpur, Malaysia

## Abstract

The anion in the title compound, (C_7_H_11_N_2_)[SnBr_2_(CH_3_)_2_(C_6_H_4_Br)], is five-coordinate within a distorted *trans*-C_3_SnBr_2_ trigonal–bipyramidal geometry. The cation and anion are linked by an N—H⋯Br hydrogen bond.

## Related literature

For the crystal structure of 4-(dimethyl­amino)­pyridinium dibromidotriphenyl­stannate(IV), see: Norhafiza *et al.* (2008 ▶).
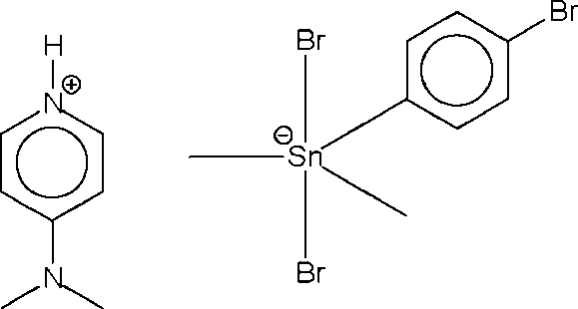


## Experimental

### 

#### Crystal data

(C_7_H_11_N_2_)[SnBr_2_(CH_3_)_2_(C_6_H_4_Br)]
*M* *_r_* = 587.76
Triclinic, 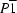

*a* = 7.3397 (1) Å
*b* = 11.1034 (2) Å
*c* = 12.2270 (2) Å
α = 100.038 (1)°
β = 102.472 (1)°
γ = 94.679 (1)°
*V* = 950.65 (3) Å^3^
*Z* = 2
Mo *K*α radiation
μ = 7.64 mm^−1^
*T* = 100 (2) K
0.30 × 0.20 × 0.02 mm

#### Data collection

Bruker SMART APEX diffractometer
Absorption correction: multi-scan (*SADABS*; Sheldrick, 1996 ▶) *T*_min_ = 0.208, *T*_max_ = 0.862
8826 measured reflections
4346 independent reflections
3718 reflections with *I* > 2σ(*I*)
*R*_int_ = 0.029

#### Refinement

*R*[*F* ^2^ > 2σ(*F* ^2^)] = 0.031
*wR*(*F* ^2^) = 0.096
*S* = 1.13
4346 reflections
194 parameters
H-atom parameters constrained
Δρ_max_ = 1.07 e Å^−3^
Δρ_min_ = −1.24 e Å^−3^

### 

Data collection: *APEX2* (Bruker, 2007 ▶); cell refinement: *SAINT* (Bruker, 2007 ▶); data reduction: *SAINT*; program(s) used to solve structure: *SHELXS97* (Sheldrick, 2008 ▶); program(s) used to refine structure: *SHELXL97* (Sheldrick, 2008 ▶); molecular graphics: *X-SEED* (Barbour, 2001 ▶); software used to prepare material for publication: *publCIF* (Westrip, 2008 ▶).

## Supplementary Material

Crystal structure: contains datablocks global, I. DOI: 10.1107/S1600536808032248/tk2315sup1.cif

Structure factors: contains datablocks I. DOI: 10.1107/S1600536808032248/tk2315Isup2.hkl

Additional supplementary materials:  crystallographic information; 3D view; checkCIF report

## Figures and Tables

**Table 1:** Hydrogen-bond geometry (Å, °)

*D*—H⋯*A*	*D*—H	H⋯*A*	*D*⋯*A*	*D*—H⋯*A*
N1—H1⋯Br1	0.88	2.56	3.319 (3)	146

**Fig. 1.**
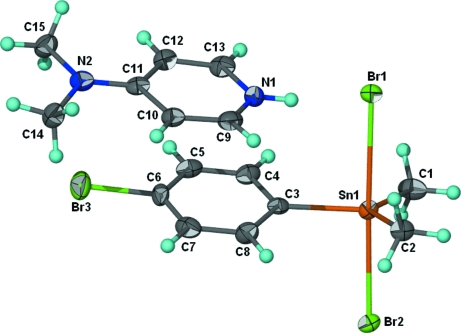
Thermal ellipsoid plot (Barbour, 2001) of [C7H11N2][SnBr2(CH3)2(C6H4Br)] at the 70% probability level. Hydrogen atoms are drawn as spheres of arbitrary radius.

## supplementary crystallographic information

### Experimental

Bis(4-bromophenyl)dimethyltin (0.10 g, 0.2 mmol) [which was prepared by the reaction between dimethyltin dichloride and 4-bromophenylmagnesium bromide] and 4-dimethylaminopyridine hydrobromide perbromide (0.08 g, 0.2 mmol) were heated in chloroform (100 ml) for 3 h. The solution was filtered and the solvent allow to evaporate to give colorless crystals.

### Refinement

Carbon-bound H-atoms were placed in calculated positions (C—H 0.95 to 0.98 Å) and were included in the refinement in the riding model approximation, with *U*(H) set to 1.2–1.5*U*_eq_(C). The ammonium H-atom was similarly treated [(N—H 0.88 Å; *U*(H) = 1.2*U*_eq_(N)]. The final difference Fourier map had a large peak at 1 Å and a deep hole at about 1 Å from the Sn1 atom.

### Crystal data

**Table:**

(C_7_H_11_N_2_)[SnBr_2_(CH_3_)_2_(C_6_H_4_Br)]	*Z* = 2
*M**_r_* = 587.76	*F*(000) = 560
Triclinic, *P*1	*D*_x_ = 2.053 Mg m^−^^3^
Hall symbol: -P 1	Mo *K*α radiation, λ = 0.71073 Å
*a* = 7.3397 (1) Å	Cell parameters from 5297 reflections
*b* = 11.1034 (2) Å	θ = 2.3–28.3°
*c* = 12.2270 (2) Å	µ = 7.64 mm^−^^1^
α = 100.038 (1)°	*T* = 100 K
β = 102.472 (1)°	Plate, colourless
γ = 94.679 (1)°	0.30 × 0.20 × 0.02 mm
*V* = 950.65 (3) Å^3^	

### Data collection

**Table:**

Bruker SMART APEX diffractometer	4346 independent reflections
Radiation source: fine-focus sealed tube	3718 reflections with *I* > 2σ(*I*)
graphite	*R*_int_ = 0.029
φ and ω scans	θ_max_ = 27.5°, θ_min_ = 1.7°
Absorption correction: multi-scan (SADABS; Sheldrick, 1996)	*h* = −9→9
*T*_min_ = 0.208, *T*_max_ = 0.862	*k* = −14→14
8826 measured reflections	*l* = −15→15

### Refinement

**Table:**

Refinement on *F*^2^	Primary atom site location: structure-invariant direct methods
Least-squares matrix: full	Secondary atom site location: difference Fourier map
*R*[*F*^2^ > 2σ(*F*^2^)] = 0.031	Hydrogen site location: inferred from neighbouring sites
*wR*(*F*^2^) = 0.096	H-atom parameters constrained
*S* = 1.13	*w* = 1/[σ^2^(*F*_o_^2^) + (0.0496*P*)^2^ + 0.4605*P*] where *P* = (*F*_o_^2^ + 2*F*_c_^2^)/3
4346 reflections	(Δ/σ)_max_ = 0.001
194 parameters	Δρ_max_ = 1.07 e Å^−^^3^
0 restraints	Δρ_min_ = −1.24 e Å^−^^3^

### Fractional atomic coordinates and isotropic or equivalent isotropic displacement parameters (Å^2^)

**Table:**

	*x*	*y*	*z*	*U*_iso_*/*U*_eq_	
Sn1	0.38417 (3)	0.25338 (2)	0.773249 (19)	0.01548 (9)	
Br1	0.74942 (5)	0.26874 (4)	0.91216 (3)	0.01853 (11)	
Br2	0.02956 (5)	0.24575 (4)	0.63868 (3)	0.01814 (11)	
Br3	0.36372 (6)	−0.36881 (4)	0.72001 (4)	0.03322 (13)	
N1	0.8504 (5)	0.0064 (3)	0.7756 (3)	0.0221 (7)	
H1	0.8438	0.0862	0.7872	0.027*	
N2	0.8761 (5)	−0.3667 (3)	0.7213 (2)	0.0199 (7)	
C1	0.3068 (6)	0.3599 (4)	0.9145 (3)	0.0251 (9)	
H1A	0.1740	0.3357	0.9112	0.038*	
H1B	0.3263	0.4475	0.9115	0.038*	
H1C	0.3845	0.3456	0.9859	0.038*	
C2	0.5034 (5)	0.3149 (4)	0.6446 (3)	0.0198 (8)	
H2A	0.4313	0.2714	0.5690	0.030*	
H2B	0.6343	0.2976	0.6553	0.030*	
H2C	0.4994	0.4037	0.6507	0.030*	
C3	0.3607 (5)	0.0584 (4)	0.7558 (3)	0.0164 (7)	
C4	0.3542 (5)	0.0007 (4)	0.8482 (3)	0.0208 (8)	
H4	0.3511	0.0496	0.9196	0.025*	
C5	0.3520 (5)	−0.1260 (4)	0.8384 (3)	0.0227 (8)	
H5	0.3462	−0.1637	0.9018	0.027*	
C6	0.3585 (5)	−0.1962 (4)	0.7338 (3)	0.0201 (8)	
C7	0.3613 (5)	−0.1437 (4)	0.6397 (3)	0.0238 (8)	
H7	0.3632	−0.1933	0.5685	0.029*	
C8	0.3611 (6)	−0.0170 (4)	0.6514 (3)	0.0217 (8)	
H8	0.3611	0.0195	0.5866	0.026*	
C9	0.8666 (6)	−0.0529 (4)	0.6733 (3)	0.0237 (8)	
H9	0.8744	−0.0076	0.6150	0.028*	
C10	0.8721 (5)	−0.1769 (4)	0.6517 (3)	0.0191 (8)	
H10	0.8787	−0.2176	0.5778	0.023*	
C11	0.8680 (5)	−0.2459 (4)	0.7390 (3)	0.0179 (8)	
C12	0.8551 (5)	−0.1788 (4)	0.8470 (3)	0.0205 (8)	
H12	0.8542	−0.2199	0.9089	0.025*	
C13	0.8440 (5)	−0.0552 (4)	0.8610 (3)	0.0232 (8)	
H13	0.8315	−0.0115	0.9325	0.028*	
C14	0.8600 (6)	−0.4359 (4)	0.6050 (3)	0.0236 (9)	
H14A	0.9550	−0.3986	0.5715	0.035*	
H14B	0.7343	−0.4337	0.5581	0.035*	
H14C	0.8799	−0.5217	0.6078	0.035*	
C15	0.8821 (6)	−0.4367 (4)	0.8132 (3)	0.0244 (9)	
H15A	0.9843	−0.3978	0.8788	0.037*	
H15B	0.9039	−0.5215	0.7858	0.037*	
H15C	0.7622	−0.4377	0.8362	0.037*	

### Atomic displacement parameters (Å^2^)

**Table:**

	*U*^11^	*U*^22^	*U*^33^	*U*^12^	*U*^13^	*U*^23^
Sn1	0.01676 (15)	0.01707 (15)	0.01403 (14)	0.00355 (11)	0.00520 (10)	0.00423 (10)
Br1	0.0172 (2)	0.0208 (2)	0.01645 (19)	0.00236 (15)	0.00153 (14)	0.00369 (15)
Br2	0.0158 (2)	0.0221 (2)	0.01817 (19)	0.00498 (15)	0.00386 (14)	0.00728 (15)
Br3	0.0257 (2)	0.0189 (2)	0.0537 (3)	0.00577 (17)	0.0064 (2)	0.0061 (2)
N1	0.0209 (17)	0.0167 (16)	0.0251 (16)	0.0044 (14)	−0.0002 (14)	0.0005 (13)
N2	0.0215 (17)	0.0251 (18)	0.0133 (14)	0.0041 (14)	0.0044 (13)	0.0033 (13)
C1	0.021 (2)	0.035 (2)	0.0181 (18)	0.0055 (18)	0.0048 (16)	0.0007 (17)
C2	0.0186 (19)	0.025 (2)	0.0178 (17)	0.0023 (16)	0.0069 (15)	0.0064 (15)
C3	0.0104 (17)	0.0211 (19)	0.0173 (17)	0.0010 (14)	0.0017 (14)	0.0047 (15)
C4	0.0190 (19)	0.027 (2)	0.0184 (18)	0.0039 (16)	0.0047 (15)	0.0083 (16)
C5	0.0160 (19)	0.031 (2)	0.0216 (19)	0.0047 (17)	0.0023 (15)	0.0092 (17)
C6	0.0133 (18)	0.0131 (18)	0.033 (2)	0.0031 (14)	0.0044 (16)	0.0037 (16)
C7	0.018 (2)	0.028 (2)	0.0228 (19)	0.0056 (17)	0.0033 (16)	−0.0019 (17)
C8	0.024 (2)	0.024 (2)	0.0183 (17)	0.0051 (16)	0.0055 (15)	0.0061 (16)
C9	0.020 (2)	0.028 (2)	0.0218 (19)	0.0023 (17)	0.0000 (16)	0.0076 (17)
C10	0.0155 (18)	0.024 (2)	0.0183 (17)	0.0041 (15)	0.0034 (15)	0.0038 (15)
C11	0.0113 (17)	0.025 (2)	0.0187 (17)	0.0056 (15)	0.0047 (14)	0.0048 (15)
C12	0.020 (2)	0.024 (2)	0.0188 (18)	0.0052 (16)	0.0057 (15)	0.0048 (16)
C13	0.0167 (19)	0.029 (2)	0.0241 (19)	0.0082 (17)	0.0055 (16)	0.0022 (17)
C14	0.033 (2)	0.022 (2)	0.0182 (18)	0.0088 (18)	0.0084 (17)	0.0027 (16)
C15	0.030 (2)	0.024 (2)	0.0202 (18)	0.0065 (18)	0.0052 (17)	0.0087 (16)

### Geometric parameters (Å, °)

**Table:**

Sn1—C1	2.127 (4)	C4—H4	0.9500
Sn1—C2	2.139 (3)	C5—C6	1.390 (6)
Sn1—C3	2.127 (4)	C5—H5	0.9500
Sn1—Br1	2.8211 (4)	C6—C7	1.380 (6)
Sn1—Br2	2.7486 (4)	C7—C8	1.390 (6)
Br3—C6	1.899 (4)	C7—H7	0.9500
N1—C9	1.343 (5)	C8—H8	0.9500
N1—C13	1.351 (5)	C9—C10	1.361 (6)
N1—H1	0.8800	C9—H9	0.9500
N2—C11	1.329 (5)	C10—C11	1.422 (5)
N2—C15	1.469 (5)	C10—H10	0.9500
N2—C14	1.469 (5)	C11—C12	1.426 (5)
C1—H1A	0.9800	C12—C13	1.364 (6)
C1—H1B	0.9800	C12—H12	0.9500
C1—H1C	0.9800	C13—H13	0.9500
C2—H2A	0.9800	C14—H14A	0.9800
C2—H2B	0.9800	C14—H14B	0.9800
C2—H2C	0.9800	C14—H14C	0.9800
C3—C4	1.400 (5)	C15—H15A	0.9800
C3—C8	1.400 (5)	C15—H15B	0.9800
C4—C5	1.390 (6)	C15—H15C	0.9800
			
C1—Sn1—C2	128.9 (2)	C7—C6—C5	121.7 (4)
C1—Sn1—C3	118.8 (2)	C7—C6—Br3	119.2 (3)
C2—Sn1—C3	112.1 (1)	C5—C6—Br3	119.1 (3)
C1—Sn1—Br2	90.82 (11)	C6—C7—C8	118.7 (3)
C3—Sn1—Br2	92.78 (10)	C6—C7—H7	120.7
C2—Sn1—Br2	89.98 (10)	C8—C7—H7	120.7
C1—Sn1—Br1	88.38 (11)	C7—C8—C3	121.8 (4)
C3—Sn1—Br1	88.88 (10)	C7—C8—H8	119.1
C2—Sn1—Br1	89.39 (10)	C3—C8—H8	119.1
Br1—Sn1—Br2	178.33 (1)	N1—C9—C10	121.1 (4)
C9—N1—C13	120.7 (3)	N1—C9—H9	119.4
C9—N1—H1	119.7	C10—C9—H9	119.4
C13—N1—H1	119.7	C9—C10—C11	120.4 (3)
C11—N2—C15	121.9 (3)	C9—C10—H10	119.8
C11—N2—C14	119.9 (3)	C11—C10—H10	119.8
C15—N2—C14	117.9 (3)	N2—C11—C10	122.0 (3)
Sn1—C1—H1A	109.5	N2—C11—C12	121.5 (3)
Sn1—C1—H1B	109.5	C10—C11—C12	116.5 (4)
H1A—C1—H1B	109.5	C13—C12—C11	119.6 (3)
Sn1—C1—H1C	109.5	C13—C12—H12	120.2
H1A—C1—H1C	109.5	C11—C12—H12	120.2
H1B—C1—H1C	109.5	N1—C13—C12	121.6 (3)
Sn1—C2—H2A	109.5	N1—C13—H13	119.2
Sn1—C2—H2B	109.5	C12—C13—H13	119.2
H2A—C2—H2B	109.5	N2—C14—H14A	109.5
Sn1—C2—H2C	109.5	N2—C14—H14B	109.5
H2A—C2—H2C	109.5	H14A—C14—H14B	109.5
H2B—C2—H2C	109.5	N2—C14—H14C	109.5
C4—C3—C8	117.4 (4)	H14A—C14—H14C	109.5
C4—C3—Sn1	122.0 (3)	H14B—C14—H14C	109.5
C8—C3—Sn1	120.5 (3)	N2—C15—H15A	109.5
C5—C4—C3	121.9 (4)	N2—C15—H15B	109.5
C5—C4—H4	119.1	H15A—C15—H15B	109.5
C3—C4—H4	119.1	N2—C15—H15C	109.5
C4—C5—C6	118.4 (4)	H15A—C15—H15C	109.5
C4—C5—H5	120.8	H15B—C15—H15C	109.5
C6—C5—H5	120.8		
			
C1—Sn1—C3—C4	−20.4 (4)	C6—C7—C8—C3	0.9 (6)
C2—Sn1—C3—C4	156.0 (3)	C4—C3—C8—C7	−2.1 (6)
Br2—Sn1—C3—C4	−112.9 (3)	Sn1—C3—C8—C7	174.7 (3)
Br1—Sn1—C3—C4	67.2 (3)	C13—N1—C9—C10	1.7 (6)
C1—Sn1—C3—C8	162.9 (3)	N1—C9—C10—C11	−2.4 (6)
C2—Sn1—C3—C8	−20.6 (3)	C15—N2—C11—C10	176.6 (4)
Br2—Sn1—C3—C8	70.5 (3)	C14—N2—C11—C10	−9.5 (6)
Br1—Sn1—C3—C8	−109.5 (3)	C15—N2—C11—C12	−3.3 (6)
C8—C3—C4—C5	1.3 (6)	C14—N2—C11—C12	170.6 (3)
Sn1—C3—C4—C5	−175.5 (3)	C9—C10—C11—N2	−179.0 (4)
C3—C4—C5—C6	0.7 (6)	C9—C10—C11—C12	0.9 (5)
C4—C5—C6—C7	−2.0 (6)	N2—C11—C12—C13	−178.9 (4)
C4—C5—C6—Br3	178.0 (3)	C10—C11—C12—C13	1.2 (5)
C5—C6—C7—C8	1.2 (6)	C9—N1—C13—C12	0.5 (6)
Br3—C6—C7—C8	−178.8 (3)	C11—C12—C13—N1	−1.9 (6)

### Hydrogen-bond geometry (Å, °)

**Table:**

*D*—H···*A*	*D*—H	H···*A*	*D*···*A*	*D*—H···*A*
N1—H1···Br1	0.88	2.56	3.319 (3)	146
